# Heterotrophy as a tool to overcome the long and costly autotrophic scale-up process for large scale production of microalgae

**DOI:** 10.1038/s41598-019-50206-z

**Published:** 2019-09-26

**Authors:** A. Barros, H. Pereira, J. Campos, A. Marques, J. Varela, J. Silva

**Affiliations:** 1 ALLMICROALGAE, Natural Products S.A., Avenida Eng° Duarte Pacheco, 19 – 9°, 1070-100 Lisboa, Portugal; 2 CCMAR - Centre of Marine Sciences, University of Algarve, Gambelas, 8005-139 Faro, Portugal

**Keywords:** Industrial microbiology, Applied microbiology

## Abstract

Industrial scale-up of microalgal cultures is often a protracted step prone to culture collapse and the occurrence of unwanted contaminants. To solve this problem, a two-stage scale-up process was developed – heterotrophically *Chlorella vulgaris* cells grown in fermenters (1^st^ stage) were used to directly inoculate an outdoor industrial autotrophic microalgal production unit (2^nd^ stage). A preliminary pilot-scale trial revealed that *C. vulgaris* cells grown heterotrophically adapted readily to outdoor autotrophic growth conditions (1-m^3^ photobioreactors) without any measurable difference as compared to conventional autotrophic inocula. Biomass concentration of 174.5 g L^−1^, the highest value ever reported for this microalga, was achieved in a 5-L fermenter during scale-up using the heterotrophic route. Inocula grown in 0.2- and 5-m^3^ industrial fermenters with mean productivity of 27.54 ± 5.07 and 31.86 ± 2.87 g L^−1^ d^−1^, respectively, were later used to seed several outdoor 100-m^3^ tubular photobioreactors. Overall, all photobioreactor cultures seeded from the heterotrophic route reached standard protein and chlorophyll contents of 52.18 ± 1.30% of DW and 23.98 ± 1.57 mg g^−1^ DW, respectively. In addition to providing reproducible, high-quality inocula, this two-stage approach led to a 5-fold and 12-fold decrease in scale-up time and occupancy area used for industrial scale-up, respectively.

## Introduction

Industrial production of microalgal biomass is one of the most promising approaches to supplying next-generation foods, feeds, and biofuels. Concomitantly, it can be coupled with the capture of CO_2_ released by emitting industries and the recycling of nutrients present in wastewaters^1,2^. Large-scale microalgal production has been tested since the 1950’s, given its high potential as cell-factories^3,4^. Microalgal biomass contains a wide range of molecules and potential products that can be obtained in the form of carbohydrates, lipids, pigments, minerals, vitamins and enzymes^4–6^. However, the high production costs of biomass production restrict microalgal ventures to the commercialization of high value products for niche markets (e.g., food supplements).

The scale-up of cultures to be grown in industrial photobioreactors (PBRs) presents a significant cost in the whole microalgal production pipeline. In addition, the production of high value biomass requires the constant production of high quality inocula to seed industrial reactors. Currently, the scale-up of autotrophic cultures is a time-consuming process occupying a significant area of the industrial production plants. Although most microalgae are photoautotrophic organisms, several strains have retained the capacity to use different carbon sources to grow heterotrophically, using organic compounds as the sole carbon source. The heterotrophic production of microalgae has consistently improved growth performance and cell concentrations compared to photoautotrophic production^7,8^. Conversely, heterotrophic cultivation of microalgae can increase the production costs, due to the need of an organic carbon source, and lead to lower protein and pigment contents, which can decrease the biomass market value^9^. However, the use of this process to exclusively seed industrial-scale systems, either PBRs or raceways, has the potential to deliver reproducible high-quality inocula, decreasing significantly the operational time and occupancy area of PBRs used in culture scale-up. A successful industrial production of heterotrophic biomass of microalgae has been achieved by Solazyme Bunge renewable oils (recently acquired by Corbion) in Brazil for oil production in 625-m^3^ fermenters.

A possible solution for improving microalgal scale-up is to combine both modes of microalgal cultivation into an efficient two-stage production. This approach relies on heterotrophic growth to obtain highly concentrated inocula for reactors operating under photoautotrophy. Such sequential systems have been tested at lab-scale, for instance, with the excavate *Euglena gracilis*^10^ and the chlorophytes *Chlorella pyrenoidosa*^9^ and *Haematococcus pluvialis*^11^.

Here we report that heterotrophically grown *Chlorella vulgaris* inocula can be used to seed 1-m^3^ PBRs for autotrophic cultivation without any measurable lag phase caused by the metabolic shift to the new trophic conditions. The full potential of this approach was demonstrated in a second experiment where heterotrophically grown inocula in industrial fermenters were used successfully to seed industrial PBR (800 m^3^). To the authors’ knowledge, this is the first report discussing the use of heterotrophically grown microalgae as inoculum for PBRs in an industrial facility. Moreover, the implementation of this mixed two-stage approach allowed the authors to obtain the highest cell concentration ever reported for *C. vulgaris*.

## Results and Discussion

### Autotrophic vs. heterotrophic scale-up route

**Figure 1:**
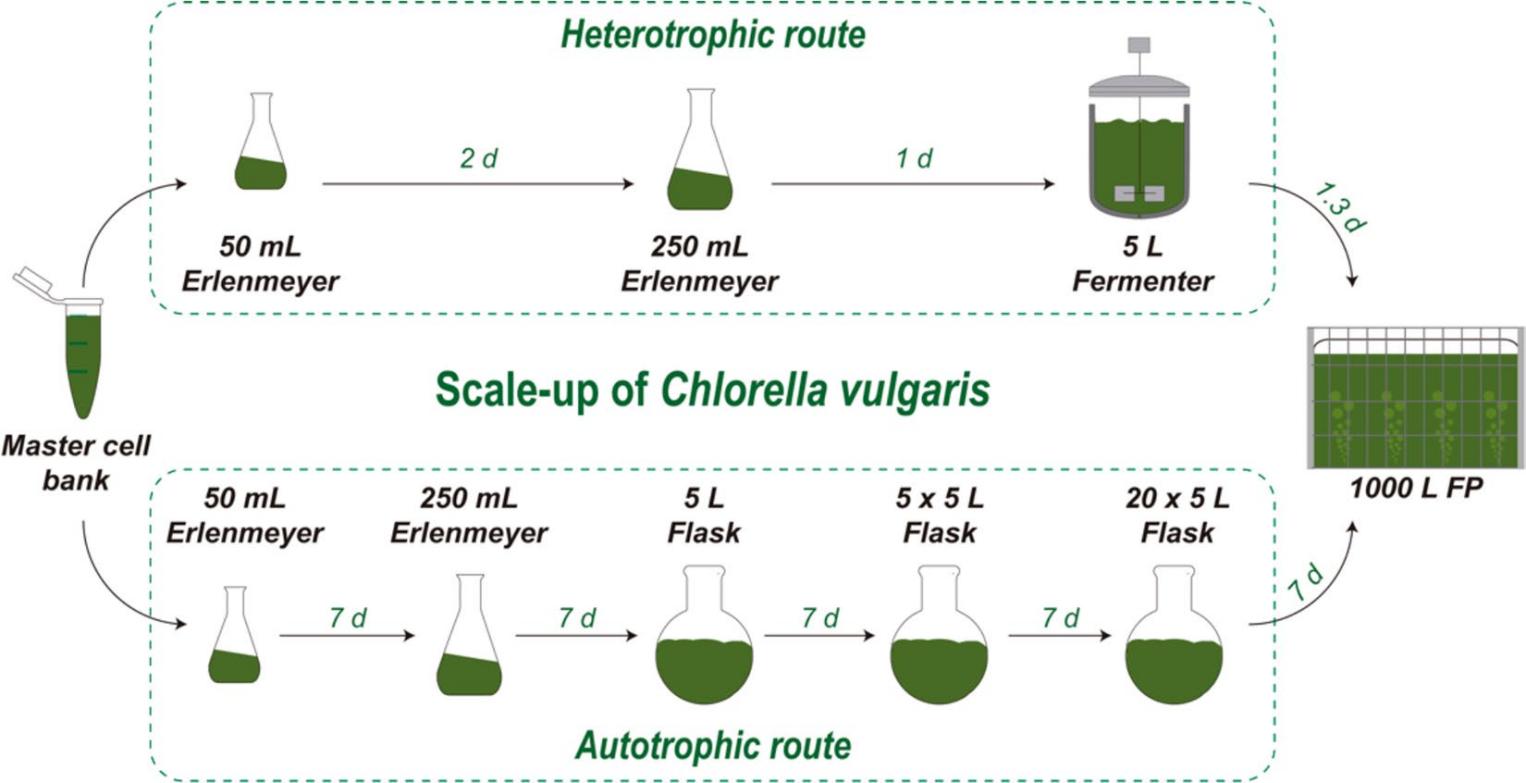
Preliminary scale-up using heterotrophically grown *Chlorella vulgaris* up to a 5-L fermenter (heterotrophic route). This procedure was compared to the standard scale-up process whose inoculum corresponded to *C. vulgaris* cells cultivated photoautotrophically in flasks with a maximum volume of 5 L (autotrophic route). Either inoculum was used to seed 1 m^3^ flat panel (FP) photobioreactors operated outdoors under photoautotrophic conditions. The culture volumes (litres) and the duration (days) of each scale-up step are indicated.

**Figure 2:**
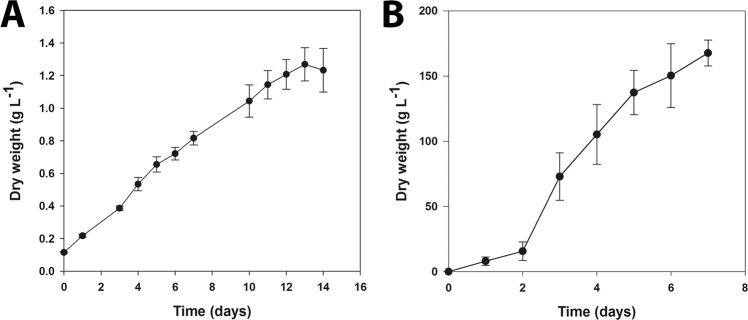
Auto- (**A**) and heterotrophic (**B**) growth of *C. vulgaris* grown in 5-L production systems. Results are expressed as the mean of dry weight and corresponding standard deviation obtained from three independent replicates.

To test the impact of the trophic shift on growth, hetero- and autotrophically cultivated *C. vulgaris* inocula were used to seed 1-m^3^ flat panels (FP) operated under photoautotrophic conditions, the first step of the production process that is carried out outdoors (Fig. 1). The growth curves of cells cultivated at the 5-L scale-up stage preceding the FP inoculation are shown in Fig. 2. Photoautotrophic cultures reached the stationary phase within 13 days with a maximum dry weight (DW) of 1.27 g L^−1^ (Fig. 2A), displaying a growth performance similar to the values previously reported for this strain^12,13^. However, in order to keep the photoautotrophically grown cells at the maximum growth rate, cultures were commonly transferred to the next scale-up stage every 7 days, until the necessary amount of inoculum was reached in order to seed the 1-m^3^ FPs. The autotrophic scale-up route, from the master cell aliquot until the seeding of the FPs, usually took 35 days (Fig. 1). Conversely, the seeding of FPs with inocula obtained via the heterotrophic route was carried out in 4.3 days, which represented an 8-fold decrease in scale-up time. In fact, heterotrophically grown inocula led to a 100-fold decrease in the volume required to start a FP, as compared to inocula obtained using autotrophic conditions. Additionally a biomass concentration of 174.5 g L^−1^ was obtained during this 5 L heterotrophic scale-up phase which is, to the authors knowledge^14,15^, the highest biomass concentration ever reported for *C. vulgaris* (Fig. 2B). In these conditions, the average growth rate and productivity were 1.05 ± 0.22 d^−1^ and 27.3 ± 6.8 g L^−1^ d^−1^, respectively. Although it was recently reported that biomass concentrations of 255 g L^−1^ can be achieved with *Auxenochlorella protothecoides* using also 5-L fermenters^16^, the final biomass productivity is similar to the values here reported.

Upon the inoculation of several 1-m^3^ FPs with inocula grown under either auto- or heterotrophy, the biomass productivities observed under photoautotrophy were, respectively, 0.11 ± 0.02 and 0.10 ± 0.01 g L^−1^ d^−1^ (Table 1). Higher productivities (0.3–0.6 g L^−1^ d^−1^) were previously reported for *C. vulgaris* in outdoor conditions; however, 2^nd^ generation Green Wall Panels (GWP-II^®^) PBRs were used, which had a lower light path and volume^17^ (10 L). The mean growth rates were 0.80 ± 0.30 and 0.60 ± 0.29 d^−1^ for the FPs inoculated with the auto- and heterotrophic inocula, respectively. As no statistic differences were observed between FPs cultivated using inocula from either trophic routes (*p* > 0.05; *n* = 29), this strongly suggests that the adaptation caused by the metabolic shift from transferring the heterotrophic inocula to outdoor autotrophic conditions had no measurable impact on the final productivity of the scale-up process.

**Table 1:** Mean and maximum biomass productivities and growth rates of flat panels inoculated from autotrophic and heterotrophic origins (*n* = 29).

Inoculum origin	Mean productivity (g L^−1^ d^−1^)	Max productivity (g L^−1^ d^−1^)	Mean growth rate (d^−1^)	Max growth rate (d^−1^)
Autotrophic	0.11 ± 0.02^a^	0.15	0.80 ± 0.30^a^	1.28
Heterotrophic	0.10 ± 0.01^a^	0.12	0.60 ± 0.29^a^	1.24

Same letters in superscript after the values denote there was no significant statistical differences (*p* > 0.05) between values on the same column.

### Two-stage industrial cultivation

**Figure 3:**
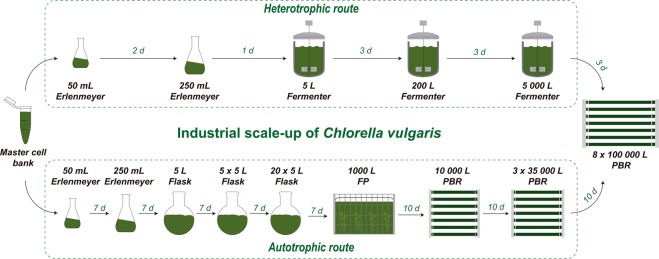
Industrial scale-up using heterotrophically grown *Chlorella vulgaris* up to a 5000-L fermenter (heterotrophic route). This procedure enabled the direct seeding of eight 100-m^3^ industrial photobioreactors, instead of the standard scale-up procedure using photoautotrophically grown inocula (autotrophic route), decreasing scale-up time and the area of the production plant dedicated to the scale-up process.

**Figure 4:**
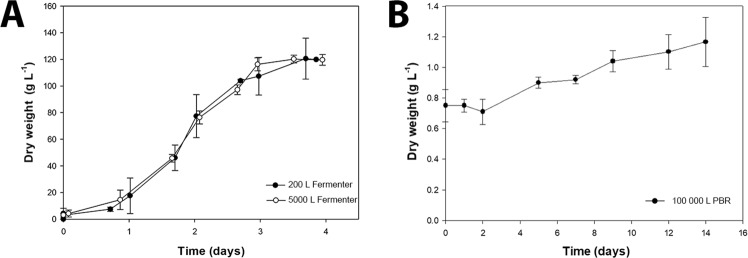
*Chlorella vulgaris* AGF002 cultivated heterotrophically in 200 and 5000-L industrial scale fermenters (*n* = 4) (**A**) or grown autotrophically in 100-m^3^ photobioreactors upon transfer from heterotrophic conditions (*n* = 8) (**B**). Values are the mean and corresponding standard deviation of four and eight replicates, respectively.

In a second experiment, the two-stage approach was further explored by scaling up the heterotrophic inocula for industrial *C. vulgaris* biomass production in order to directly seed PBRs with 100 m^3^ of volume (Fig. 3). In this way, if the preliminary results obtained in the first experiment were confirmed, further decrease in time and area needed for inoculum production in this industrial facility would become feasible. Therefore, in the first step of the process, cultures obtained from the 5-L fermenter were used to seed a 200-L scale-up fermenter, which was subsequently employed to inoculate a 5000-L industrial fermenter (Fig. 3). The 200- and 5000-L fermenters displayed similar final DW (≈120 g L^−1^; Fig. 4A), which might be explained by the similar mean productivities and growth rates (*p* > 0.05) obtained (Table 2).

**Table 2:** Mean and maximum biomass productivities and growth rates of industrial 200 and 5000 L fermenters.

Reactor volume (L)	Mean productivity (g L^−1^ d^−1^)	Maximum productivity (g L^−1^ d^−1^)	Mean growth rate (d^−1^)	Maximum growth rate (d^−1^)
200	27.54 ± 5.07^a^	32.56	0.92 ± 0.11^a^	2.44
5000	31.86 ± 2.87^a^	34.82	0.88 ± 0.26^a^	2.32

Same letters in superscript after the values denote there was no significant statistical differences (*p* > 0.05) between values on the same column.

Accordingly, the maximum biomass productivities and maximum growth rates were 32.56 and 34.82 g L^−1^ d^−1^ and 2.44 and 2.32 d^−1^, respectively. Although the final biomass concentration obtained in both industrial fermenters are lower than those previously obtained with the 5-L, it is still higher than those previously reported for highly concentrated cultures of heterotrophically grown *Chlorella vulgaris*^18^, *Chlorella pyrenoidosa*^19^, *Auxenochlorella protothecoides*^20^, and *Coelastrum microsporum*^21^ (formerly known as *Chlorella regularis*), among others.

In the second step of the two-stage growth, cultures from a 5000-L fermenter were transferred to autotrophic industrial 100-m^3^ PBRs (Fig. 4B; *n* = 8). The PBRs were seeded using heterotrophic inocula to start with an initial DW of 0.75 g L^−1^ and cultures were grown for 14 days. The onset of an apparent lag phase of two days without any growth was observed, followed by a phase of steady growth until a final DW of 1.09 ± 0.16 g L^−1^ was reached.

Overall, the heterotrophic scale-up route employed to seed industrial PBRs lasted 12 days, using an occupancy area of about 250 m^2^. This area corresponds to an industrial fermentation setup including laboratory, fermenters and utilities. On the other hand, the autotrophic route spanned 65 days using an occupancy area of approximately 2885 m^2^ to obtain eight 100-m^3^ PBRs with an initial concentration of 0.5 g L^−1^ DW. This scale-up route is based on the dilution of 1.5 g L^−1^ autotrophic inocula as described in Fig. 3. Hence, this corresponds to a 12- and 5-fold decrease in occupancy area and the duration of the scale-up process, respectively. This economy of scale has been translated into significantly lower production costs, as the scale-up is considered to be an unproductive process.

### Protein and chlorophyll build-up

**Figure 5:**
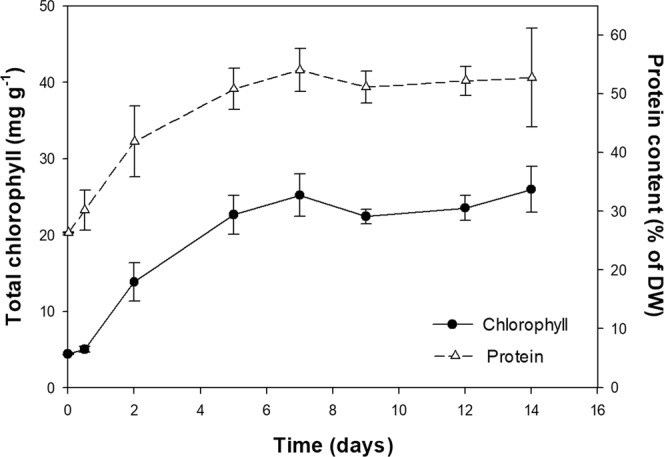
Total chlorophyll and protein content of the industrial autotrophic *Chlorella vulgaris* cultures from heterotrophic inocula. Error bars represent the standard deviation calculated from eight replicates (*n* = 8).

Finally, in order to better understand the impact of this novel scale-up procedure on the quality of the biomass, the chlorophyll and protein build-up in the 100-m^3^ industrial PBRs was assessed throughout the growth period. The chlorophyll content of the inoculum obtained from heterotrophic origin was approximately 5 mg g^−1^ DW. This content gradually increased within the first 7 days of autotrophic growth. Longer cultivation time did not result in higher concentrations of total chlorophyll, which reached a plateau of 23.98 ± 1.57 mg g^−1^ DW (Fig. 5). Chlorophyll content between 10 and 38 mg g^−1^ DW have been reported for *C. vulgaris* grown photoautotrophically^22–24^. Indeed, a comparable hetero- to autotrophic sequential cultivation has yielded a similar chlorophyll accumulation pattern with a maximum of 36 mg g^−1^ DW of total chlorophyll^19^. The protein content of the cultures transferred to autotrophic conditions increased by 70%, reaching a plateau of 52.18 ± 1.30% of DW (Fig. 5). These protein levels are within the 50–60% range previously reported for autotrophically grown *C. vulgaris*^9,22^. Furthermore, as chlorophyll and protein contents are influenced by several factors such as nitrogen concentration and light availability^22,24,25^, the authors believe that further increases in the protein and pigment contents are attainable.

## Conclusions

A scale-up procedure using heterotrophic growth to generate highly concentrated microalgal inocula to seed industrial photobioreactors can be essential to large production. This two-stage approach enables significant savings in scale-up time and footprint dedicated to the early stages of the production process. Moreover, the use of scale-up fermenters avoids the build-up of contaminants and culture collapse, being also a weather-independent operation. These advantages can lead to important savings in CAPEX (capital expenditure) and OPEX (operational expenditure). The combination of hetero- with photoautotrophic growth enables the production of microalgal biomass with pigment and protein contents that are observed in cultures grown exclusively under photoautotrophic conditions.

## Materials and Methods

### Microalgal growth

*Chlorella vulgaris* AGF002 was cultivated in auto- and heterotrophic media. The composition of the Guillard’s F2 culture medium was adapted to the local water to obtain the autotrophic media (AM), using nitrate as nitrogen source. The heterotrophic media (HM) was obtained by adding glucose as the organic carbon source with a C:N ratio of 6.7:1. The seed culture was obtained from cryopreserved cultures stored in liquid nitrogen (master cell bank - MCB). One cryovial was then scaled up to obtain the auto- and heterotrophic inocula, as shown in Fig. 1.

### Autotrophic route

Cultures were grown autotrophically in 5-L flasks during 7 to 15 days as described in Fig. 1 with AM, using a photoperiod of 24:0 (Light:Dark). The pH was maintained between 7 and 8 by periodic injection of CO_2_. Typically, using this trophic route, cultures starting at 0.2 g L^−1^ could reach biomass concentrations of 1.2 g L^−1^. At this stage, the 20 × 5 L flasks of culture (100 L) were used to inoculate a single FP.

### Heterotrophic route

The seed for heterotrophic growth was obtained sequentially in 50- and 250-mL Erlenmeyer’s, in order to reach a volume of 7 L in a bench-top fermenter (New Brunswick BioFlo^®^ CelliGen®115; Eppendorf AG, Hamburg, Germany). Temperature was maintained at 28 °C and pH at 6.5 by addition of ammonia solution (24% w/w). The bioreactor was operated in fed-batch mode as glucose was fed upon its depletion, enabling the harvest of the culture at multiple time points. Samples were collected aseptically and centrifuged at 6500 *g* for 2 min (VWR Micro Star 12, Pennsylvania, USA) for supernatant analysis or used as is for biomass concentration analysis. Throughout the growing period the air inlet flowrate was adjusted to maintain approximately 1 vvm. Accordingly, the agitation rate ranged from 100 to 1200 rpm, so that the dissolved oxygen in the medium was not a limiting factor for culture growth. Glucose concentration in the supernatant was monitored in a YSI-2900 (YSI Inc./Xylem Inc, Ohio, USA). Biomass growth was monitored by optical density measurements at 600 nm using Genesys 10S UV-Vis spectrophotometer (Thermo Fisher Scientific, Massachusetts, USA). When sufficiently concentrated, the bioreactor was harvested to inoculate one or several FPs. After harvesting the culture, fresh HM was added, and the heterotrophic process continued in semi-continuous mode.

### Cultivation in flat panels

A total of 29 FPs (1 m^3^ working volume) were used to grow 16 auto- and 13 heterotrophic inocula from 24 May to 23 July, 2016. In this time period, the average temperature (24.2 ± 2.2 °C) and radiation (316.6 ± 42.5 W m^−2^) registered in the greenhouse where the FPs are located were very stable. A biomass at a concentration of 0.12 ± 0.03 g L^−1^ and AM with a nitrogen content of 10 mM N L^−1^ were used to initiate the cultures in the FPs. The pH was maintained between 7 and 8 by timed injections of CO_2_ and continuous aeration.

### Cultivation in industrial fermenters

Industrial fermentation was performed in 200-L and 5000-L fermenters via the heterotrophic route in order to obtain inoculum for the autotrophic industrial photobioreactors. Both industrial fermenters were operated in fed-batch under the conditions previously described in section “Heterotrophic route”. When sufficiently concentrated, the 200-L culture was used to inoculate the 5000-L reactor, whose cells were then used to inoculate 8 tubular PBR with a working volume of 100-m^3^. This culture scale-up was compared to the exclusively autotrophic approach as described in Barros *et al*.^26^.

### Cultivation in industrial PBR

Eight industrial scale tubular PBR with a working volume of 100 m^3^ were inoculated with heterotrophically grown *C. vulgaris* cultures from the 5000-L industrial fermenter. These PBR are located outdoors and were grown autotrophically from 2 to 16 August, 2017 at Algafarm. The cultures were subjected to a natural circadian light:dark cycle with approximately 16 h of light per day and peak radiation of 687 ± 53 W m^−2^. Minimum and maximum temperatures ranged from 16.4 and 26.2 °C. Initial biomass and AM concentration were 0.75 ± 0.11 g L^−1^ and 10 mM N L^−1^, respectively. The pH was maintained between 5.5 and 6.5 by injection of pure CO_2_ and continuous aeration.

### Growth estimation

DW was obtained by filtration of culture samples using pre-weighed 0.7 µm GF/C 698 filters (VWR, Pennsylvania, USA) and dried at 120 °C until constant mass was obtained using a DBS 60–30 electronic moisture analyser (KERN & SOHN GmbH, Balingen - Germany). All dry weight samples were washed with demineralized water to remove growth medium salts. Whenever the previous procedure could not be carried out, a DW vs. optical density correlation developed in-house for this strain grown under different culture conditions (e.g., auto- or heterotrophic growth) was used. Biomass productivity was obtained by Eq. 1 and growth rate by Eqs 2.1 or 2.2 depending upon the use of the autotrophic or the heterotrophic routes, respectively.

1 $$P(g{L}^{-1}{d}^{-1})=\frac{(D{W}_{f}-D{W}_{i})}{({t}_{f}-{t}_{i})};$$

2.1 $${\rm{\mu }}({d}^{-1})=\frac{(D{W}_{f}-D{W}_{i})/D{W}_{i}}{({t}_{f}-{t}_{i})};$$

2.2 $${\rm{\mu }}({d}^{-1})=\frac{\mathrm{ln}(D{W}_{f}/D{W}_{i})}{({t}_{f}-{t}_{i})};$$

### Estimation of chlorophyll content

Culture samples were centrifuged at 2547 *g* for 15 min using a Hermle centrifuge (HERMLE Labortechnik GmbH, Wehingen – Germany). Pigments were extracted from the resulting pellet by bead milling in acetone. The full absorbance spectrum of the extract was obtained with a Genesys 10S UV-Vis spectrophotometer (Thermo Scientific, Massachusetts, EUA) and iteratively decomposed to the standard pigment spectra in order to obtain the total chlorophyll content.

### Determination of the protein content

The protein content of cultures was determined by elemental analysis, resorting to Vario el III (Vario EL, Elementar Analyser systeme, GmbH, Hanau, Germany), according to the procedure provided by the manufacturer. The final protein content was calculated by multiplying the percentage of nitrogen given by the elemental analysis by 6.25.

## Data Availability

Raw data is available on request.
